# Evaluating Effects of Skin Needling Treatment on Visible Changes and Elasticity of Scars Using High-Frequency Ultrasound, Cutometer^®^, and Standardized Questionnaire—Six Case Studies

**DOI:** 10.3390/jcm14155553

**Published:** 2025-08-06

**Authors:** Marta Wacewicz-Muczyńska, Dominika Chojnacka, Bogumiła Redlarska, Anna Kołodziejczak

**Affiliations:** 1 Department of Specialist Cosmetology, Medical University of Bialystok, 15-267 Białystok, Poland; bogumila.redlarska@umb.edu.pl; 2 Independent Researcher, 15-404 Białystok, Poland; d.chojnacka2920@gmail.com; 3 Department of Cosmetology and Aesthetic Dermatology, Medical University of Łódź, Muszyńskiego 1 Street, 91-151 Łódź, Poland; anna.kolodziejczak@umed.lodz.pl

**Keywords:** scar tissue, skin needling, ultrasonography, elasticity, treatment

## Abstract

**Background/Objectives**: Scars are formed from trauma to the dermis and more specifically during the wound-healing phase. Skin needling is a technique used in scar therapy which stimulates the skin to regenerate. The aim of this study was to objectively and subjectively evaluate the degree of scar reduction after skin needling treatments based on visible changes using specialized measuring devices—ultrasound, Courage & Khazaka, and standardized questionnaires. **Methods**: Six patients were enrolled. Participants were given a series of three skin needling treatments. Before and after the treatment, the participants were examined for selected skin parameters with the help of specialized measuring devices such as Courage & Khazaka and skin ultrasound. Skin firmness and elasticity and MEP and HEP skin echogenicity were taken into account. Each patient completed POSAS questionnaires on satisfaction, pain, and adverse effects. **Results**: Based on the results of the POSAS questionnaire, a significant improvement in patients’ scar evaluation was observed after the treatment. Patients reported the most noticeable improvements in parameters such as color (*p* = 0.035), stiffness (*p* = 0.009), thickness (*p* = 0.041), and irregularity (0.007). An improvement in scar elasticity was observed in all subjects after treatment. **Conclusions**: Skin needling treatment combined with the skin needling technique and post-treatment skincare is an effective method of scar therapy, and the risk of side effects or complications after a series of treatments is low.

## 1. Introduction

Cutaneous scars exert a multidimensional impact on both the psychological and somatic health of affected individuals. They can contribute to social exclusion and a diminished overall quality of daily life [1]. Epidemiological data indicate that 48.5% of adults have at least one scar, with 12.3% of men and 10.7% of women experiencing painful scars, negatively influencing their appearance and physical well-being. In addition, many patients report pain, tightness, and general discomfort in the scarred region, all of which further impair their day-to-day functioning [2,3].

Scars are natural outcomes and the final stage of the wound-healing cascade triggered by tissue injury. When this reparative process is prolonged or dysregulated, hypertrophic scars can form [4]. Such lesions are often itchy and painful and can cause deterioration of tissue elasticity, restricted mobility due to contractures, and significant functional limitations as well as esthetic defects [5].

Skin needling, also called microneedling, is a technique based on the principle of percutaneous collagen induction (PCI) therapy. This method generates micro-injuries in the dermis, triggering a wound-healing response that activates a series of growth factors, ultimately leading to collagen synthesis. Additionally, skin needling creates small channels, which increase the absorption of topically applied preparations. Skin needling can be applied using a range of devices such as manual, motorized/electrically powered like pens, stampers, and radiofrequency coupled or in combination therapy [6,7,8].

**Figure 1:**
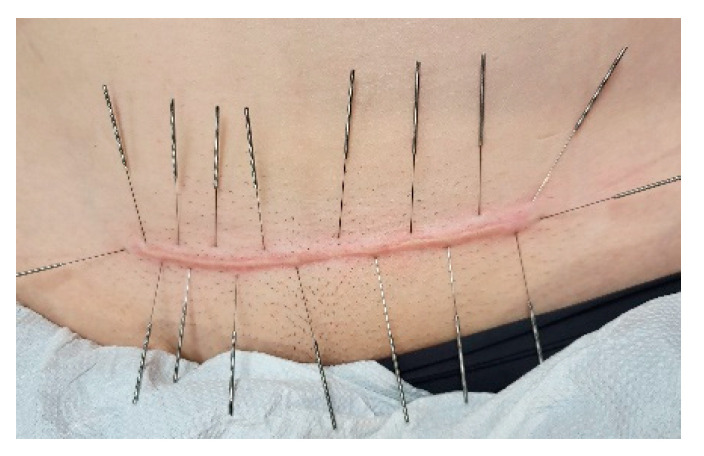
Application of dry needling in scar treatment.

Dry needling is defined as an advanced technique that uses a fine needle to pierce the skin and stimulate the muscles and connective tissues beneath, with the goal of treating neuromuscular dysfunctions and movement limitations [9,10]. Dry needling is a therapeutic intervention that involves the insertion of fine needles into myofascial trigger points or taut bands within skeletal muscles. According to the American Physical Therapy Association, it is defined as a specialized technique that utilizes a thin needle to penetrate the skin and stimulate underlying muscle and connective tissue, with the primary objective of addressing neuromuscular dysfunction and movement impairments [11]. Several theories have been proposed to explain its mechanisms and therapeutic effects. It has been suggested that the local application of needles around the scars (Figure 1) effectively facilitates the scar healing process and alleviates pain and other scar-related symptoms. Dry needling may reduce scar tissue by mechanically disrupting fibrotic adhesions and stimulating a localized inflammatory response, which promotes collagen remodeling and improved tissue elasticity. This process also enhances microcirculation, accelerates tissue healing, and modulates inflammatory and nerve activity, which helps reduce pain and improve tissue function [4,12].

Systematic scar assessment is important for monitoring the effectiveness of therapeutic interventions due to the individual patient’s needs. Scar thickness can be measured both subjectively, through clinician assessment and patient-reported outcomes, or objectively, using medical imaging methods. Traditionally, scar evaluation has relied on photographic documentation and physical examination based on expert experience. However, more recently, a variety of subjective and objective assessment tools have been developed for scar evaluation. Those methods include visual assessment using scales like the Patient and Observer Scar Assessment Scale (POSAS), ultrasonography, or objective analyzers such as the Cutometer [13,14,15].

The POSAS is a subjective assessment method that relies on both the patient’s and the clinician’s evaluation of the scar’s characteristics. It is a standardized, two-part questionnaire that evaluates scar quality across visual, tactile, and sensory domains from both the clinician’s and the patient’s perspectives. The patient component consists of seven questions about the scar, including pain, itchiness, color, stiffness, thickness, irregularity, and an overall opinion. The observer scale includes an assessment of scar vascularity, pigmentation, thickness, relief, pliability, surface area, and an overall opinion. The POSAS ranges from 1 to 10, where a score of 1 is close to normal skin, and a score of 10 is the worst scar imaginable [16]. Ultrasound imaging is a safe, non-invasive and effective method for measuring skin and scar echogenicity. Modern brightness mode (B-mode) ultrasound, particularly high-frequency (i.e., ≥20 MHz) or ultra-high-frequency (30–100 MHz) ultrasonography, allows for differentiation between the epidermis and dermis [17,18]. Ultrasonography provides objective and quantitative information, which permits clinicians to identify the current state of the scar and evaluate treatment results [19,20].

The Cutometer^®^ is a device used to assess the elasticity of the upper layer of the skin by means of negative pressure, which induces mechanical deformation of the skin. The device generates a vacuum that draws the skin into the probe’s aperture and then releases it after a specified duration [21]. Inside the probe, the depth of skin penetration is measured using a non-contact optical measurement system. The Cutometer measures the vertical deformation of the skin in millimeters when the skin is pulled by means of a controlled vacuum into the circular aperture, 2 mm in diameter, of the probe.

The aim of this study was to assess the usefulness of high-frequency ultrasound for the assessment of scars tissue and monitoring the outcomes of skin needling treatment.

## 2. Materials and Methods

### 2.1. Patients

The study group consisted of 7 adult women with a mean age of 40.16 years, presenting with atrophic or hypertrophic scars of various etiologies. The participants were volunteers who presented to the Department of Specialist Cosmetology and were qualified for the study based on information obtained through a consultation questionnaire and following a thorough assessment of the scar condition. The exclusion criteria included pregnancy, breastfeeding, a tendency to develop keloids, minority (under 18 years of age), diabetes, chronic dermatological conditions with an acute course in the treatment area (e.g., eczema, active psoriasis, atopic dermatitis), ongoing chemotherapy or radiotherapy, and the use of anticoagulant medications. One patient was excluded from the study because examination showed his scar type was keloid. All 6 patients completed the study and no one resigned from participating.

The study was approved by the Bioethics Committee of the Medical University of Bialystok (approval no. APK.002.247.2023).

### 2.2. Skin Needling Procedure

Three treatment sessions were performed at 4 to 6 week intervals. The skin needling procedure was carried out using the Soft Liner Basic device with the speed ranging from 90 to 150 insertions per second. Acupuncture needles SEIRIN J-Type with a diameter of 0.30 mm and thicknesses ranging from 0.12 to 0.30 mm were used, as well as FLAT 7-type needles (7 needles in a row, 0.30 mm thickness, 30 mm length). Additionally, in certain cases, cartridges sized 20R (twenty needles), 0.30 mm—commonly used in microneedling—were employed. Prior to the procedure, the treatment area was cleansed and disinfected. Microneedling was then performed with a duration of 15 to 20 min. The procedure of each scar was performed individually using the most adequate technique.

Upon completion of the procedure, a hydrocolloid gel (active ingredients: Panthenol, Carnosine, Silver Citrate, Allantoin) was applied to the treated area. In the case of scars located in exposed areas (e.g., a scar on the hand), a photoprotective cream containing mineral UV filters was used. The participants undergoing the procedure received written post-treatment care instructions and were thoroughly advised on how to manage and care for the treated area at home.

Prior to the series of treatments, each woman underwent skin examinations using specialized measuring devices, which were ultrasonography and the Courage & Khazaka modular device, where heads were used to measure skin elasticity and echogenicity. Before starting the treatment, for each scar, a single point was selected for measurement—identified as the most problematic area by both the patient and the investigator. Each scar was marked with a pen and photographed to enable evaluation of the same site on follow-up. All measurements were conducted 6–8 weeks before and after the last treatment in the series.

### 2.3. Patient Observer Scar Assessment Scale (POSAS)

The POSAS is a validated scar assessment tool which allows for both objective (by the observer) and subjective (by the patient) assessment of the scar’s appearance and sensations. During the series of skin needling treatment, each of the participants as well as the investigator completed the POSAS questionnaire. The patients were blinded from the observers’ scores and rated their own scars using the patient component of the POSAS. The questionnaire was completed twice—before and after (without access to previous results) the series of treatment. The same researcher and patients completed the POSAS before and after treatment.

### 2.4. Assessment of Scar Elasticity

A scientific Cutometer^®^ dual MPA 580 multi-probe system [Courage & Khazaka Electronic, Köln, Germany] was used to assess scar elasticity.

**Figure 2:**
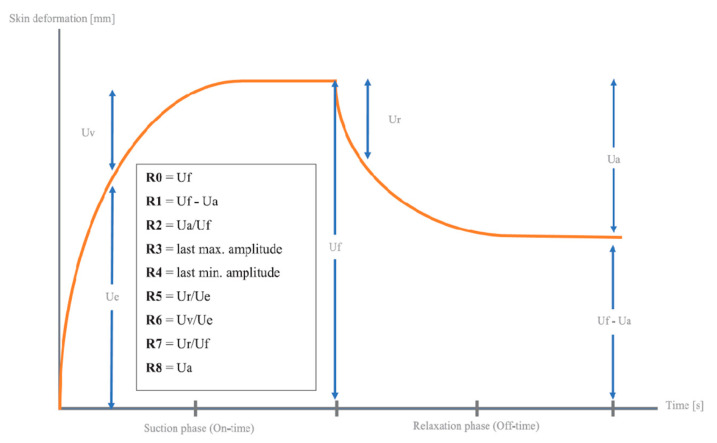
Cutometer output with R-parameters representing specific areas of skin recovery.

Measurements were conducted using Mode 1 of the device, with a vacuum pressure of 450 mbar. Both the active and passive suction durations were set to 3 s. A full curve was composed of three cycles of suction/relaxation, thus 18 s total per curve. One full trial was composed of 3 curves without removing or altering probe position during the measurement of those 3 curves. The skin’s response to negative pressure and the following release are displayed as curves in Figure 2. According to the literature, R0 is the most relevant parameter for evaluating skin defects such as scars. Among the fifteen parameters computed by the Cutometer^®^ software (MPA CTplus), R0 demonstrated the highest inter-rater reliability. It is also considered the primary parameter for scar assessment [22,23]. R0 means the behavior of the skin/scar to force (rigidity), with the maximum amplitude of the curve in mm. From these curves. a number of parameters are calculated representing different quantitative measures of mechanical skin properties such as firmness, pliability, and elasticity (Table 1). Elasticity measurements were conducted before and after treatment. According to the recommendation of Cutometer^®^ producent (Courage + Khazaka) the measurements were performed by the same researcher to ensure consistency and minimize inter-rater variability.

### 2.5. Assessment of Scar Echogenicity

The measurements were conducted using a DermaMed Dramiński ultrasound device operating at a frequency of 48 MHz, which permits the detailed visualization of the epidermis, dermis, and the upper part of the subcutaneous tissue, reaching a depth of approximately 3–4 mm [19,24].

In this study, skin echogenicity within the scarred areas was assessed before and after the therapy, and the results were compared. The structure of each scar was evaluated by calculating the proportion of bright pixels to the total number of pixels (TP—total pixels) in the selected region of the ultrasound image. After obtaining ultrasound images, thickness and echogenicity were evaluated on a personal computer using the software provided by the manufacturer of the ultrasound device. Pixel classification was defined as follows: LEP (Low Echogenic Pixels): dark pixels, range 0–30; MEP (Medium Echogenic Pixels): range 50–150; HEP (High Echogenic Pixels): range 200–255 [25,26]. For the purpose of scar evaluation, the MEP and HEP ranges (150–255) within the region of interest were analyzed.

### 2.6. Statistical Analysis

**Figure 3:**
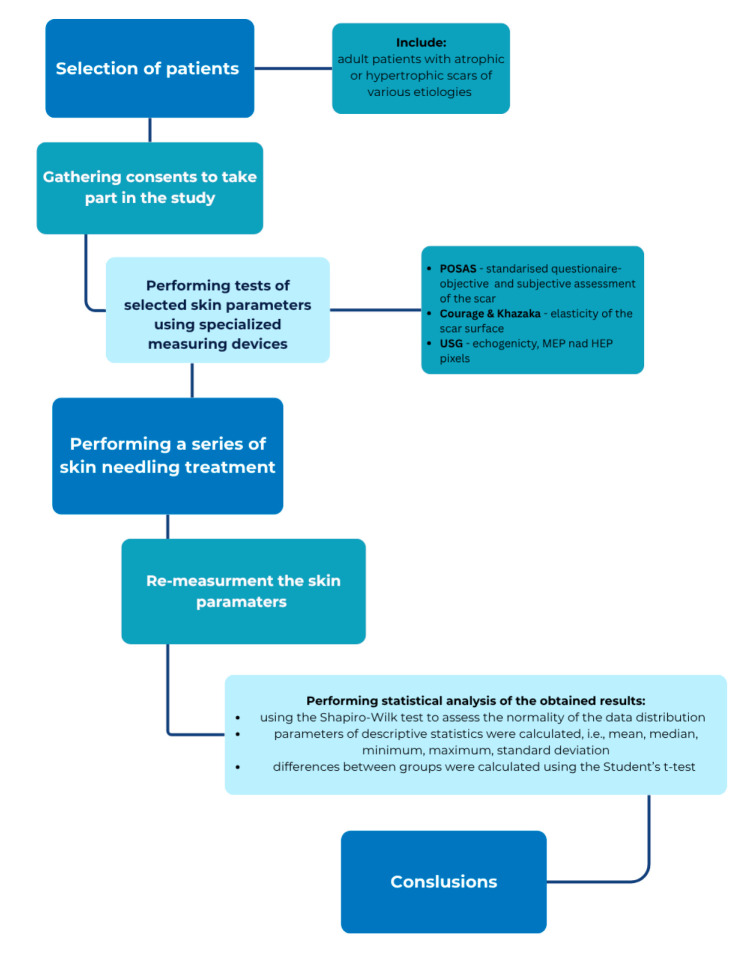
Flowchart of study course.

The results obtained were analyzed using the Statistica V 14.0. statistical program and Microsoft Office Excel 2019 software. Parameters of descriptive statistics, i.e., mean, median, minimum, maximum, and standard deviation, were also calculated. Statistical analyses were performed using the Shapiro–Wilk test to assess the normality of the data. Differences between dependent groups were tested with Student’s *t*-test and Wilcoxon test. The level of statistical significance was taken as *p* < 0.05. The entire course of the scientific experiment is shown as a flowchart (Figure 3).

## 3. Results

The characteristics of the patients and their scars are presented in Table 2. Four out of six patients had post-surgical scars, one post-burn scar, and one patient had a post-traumatic scar. The age of the scars varied and ranged from 1.5 years to even 42 years old.

### 3.1. POSAS Questionnaire

The results obtained from the objective and subjective POSAS scar assessment questionnaire, as completed by the observer and patients, are presented in Table 3.

On the patient-reported scale of the POSAS, the average total score reported for the entire study group before therapy was 46.8 points and after the treatment, the total score decreased to 25.3 points—a 45% improvement. Patients reported the most noticeable improvements in parameters such as color (*p* = 0.035), stiffness (*p* = 0.009), thickness (*p* = 0.041), and irregularity (*p* = 0.007). Among all participants. the greatest improvements were observed in patients P1 (60% improvement), P3 (64% reduction in score), and P6 (52% reduction in score).

**Figure 4:**
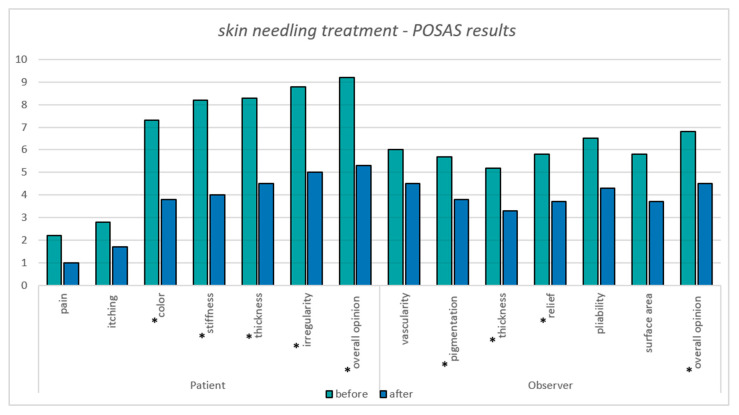
Skin needling treatment POSAS results; statistical significance, * *p* < 0.05.

The average total score completed by the observer for the entire study group before therapy was 41.8 and after the treatment, the total score decreased to 27.8 points (indicating an improvement in scar quality)—an improvement of 33%. POSAS observer attributes achieved statistical significance for pigmentation (*p* = 0.049), thickness (*p* = 0.016), relief (*p* = 0.010), and overall opinion (*p* = 0.001) (Figure 4). Among all the participants, the greatest improvement was observed in patient P6 with a score reduction of 49%, patient P2 (improvement of 40%), and P1 (37% improvement).

### 3.2. Scar Elasticity

**Figure 5:**
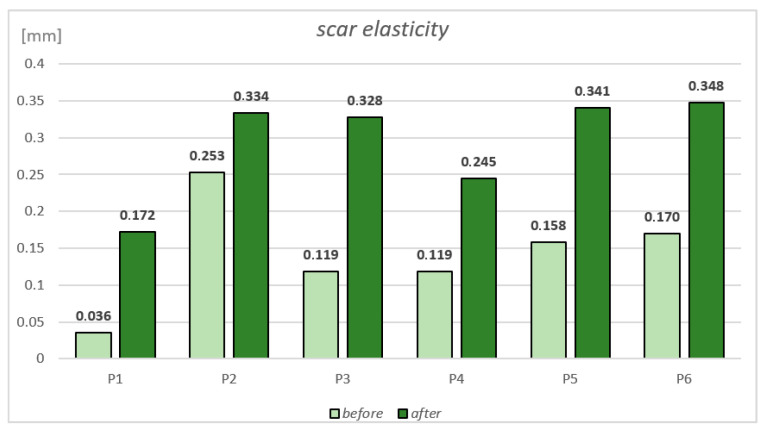
Differences in degree of scar elasticity before and after treatment among participants.

Measurements taken using the Cutometer showed that skin elasticity in the scar area improved in all patients after the treatment (Figure 5). The best results were observed in patient P1 (scar on the dorsal side of the hand), where the R0 parameter increased from 0.036 mm to 0.172 mm, resulting in significant improvement (nearly four times).

Significant improvement was also noted in patient P3 (cesarean section scar), with elasticity increasing by 176% (from 0.119 mm before to 0.328 mm after treatment).

### 3.3. Visible and Ultrasound Changes in Scar Area

**Figure 6:**
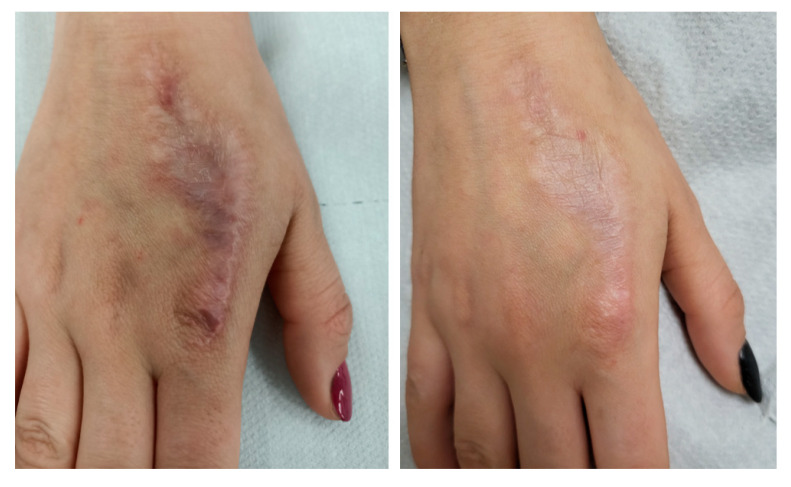

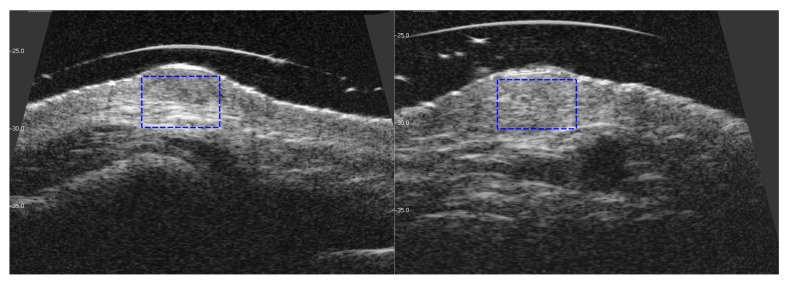
Visible ((**upper**) photo) and ultrasonographic ((**lower**) photo) changes before (**left**) and after (**right**) scar therapy of patient P1.

**Figure 7:**
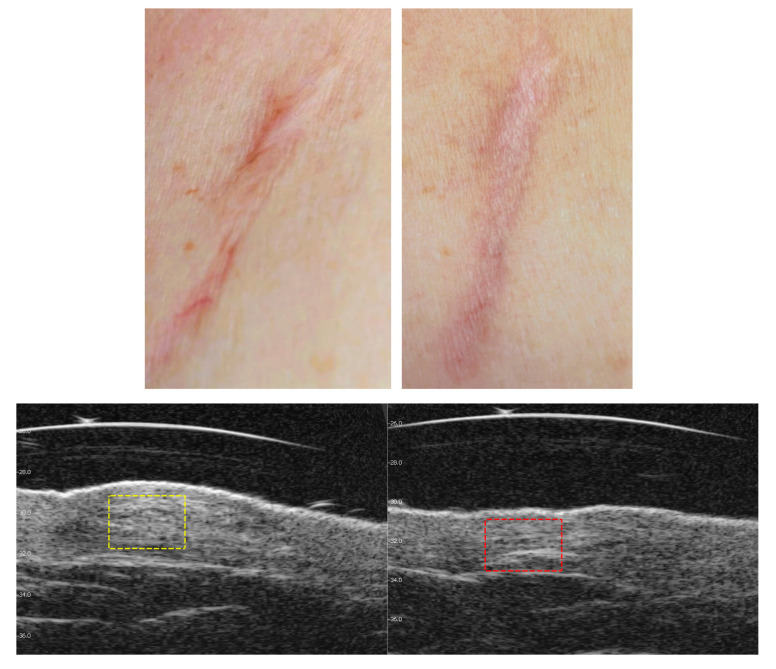
Visible ((**upper**) photo) and ultrasonographic ((**lower**) photo) changes before (**left**) and after (**right**) scar therapy of patient P2.

**Figure 8:**
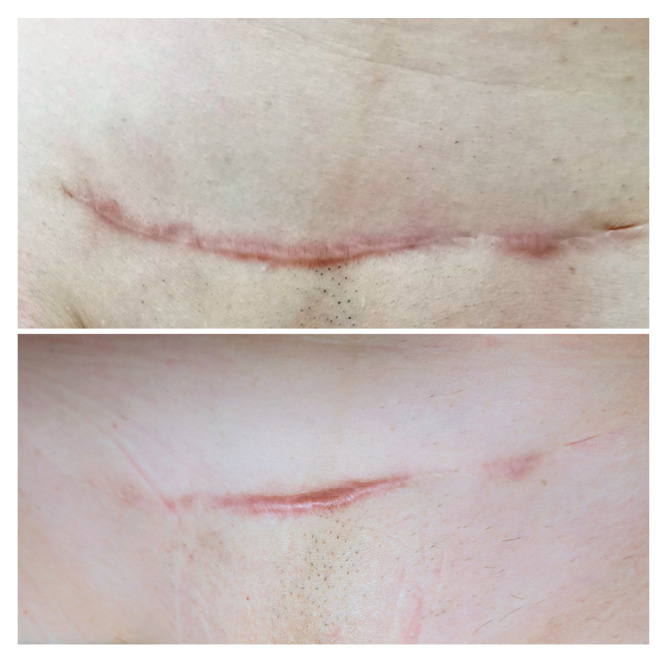

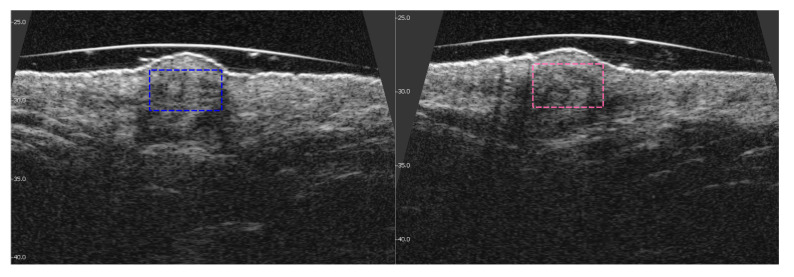
Visible ((**upper**) photo) and ultrasonographic ((**lower**) photo) changes before (**left**) and after (**right**) scar therapy of patient P3.

**Figure 9:**
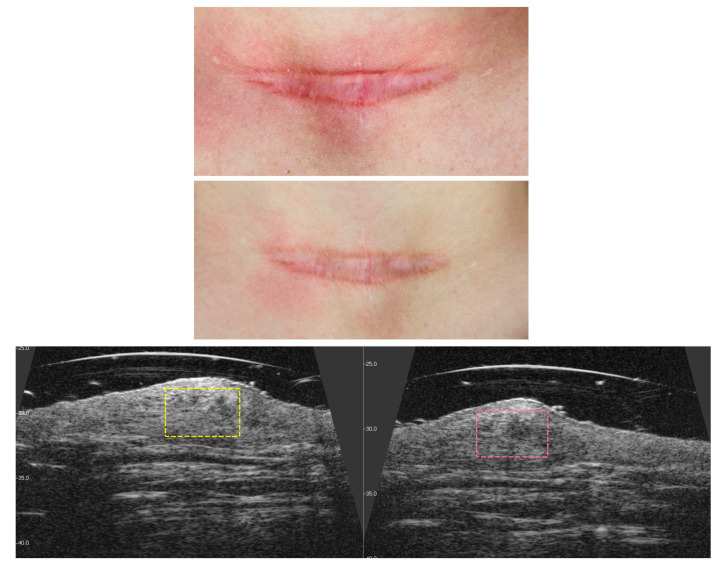
Visible ((**upper**) photo) and ultrasonographic ((**lower**) photo) changes before (**left**) and after (**right**) scar therapy of patient P4.

**Figure 10:**
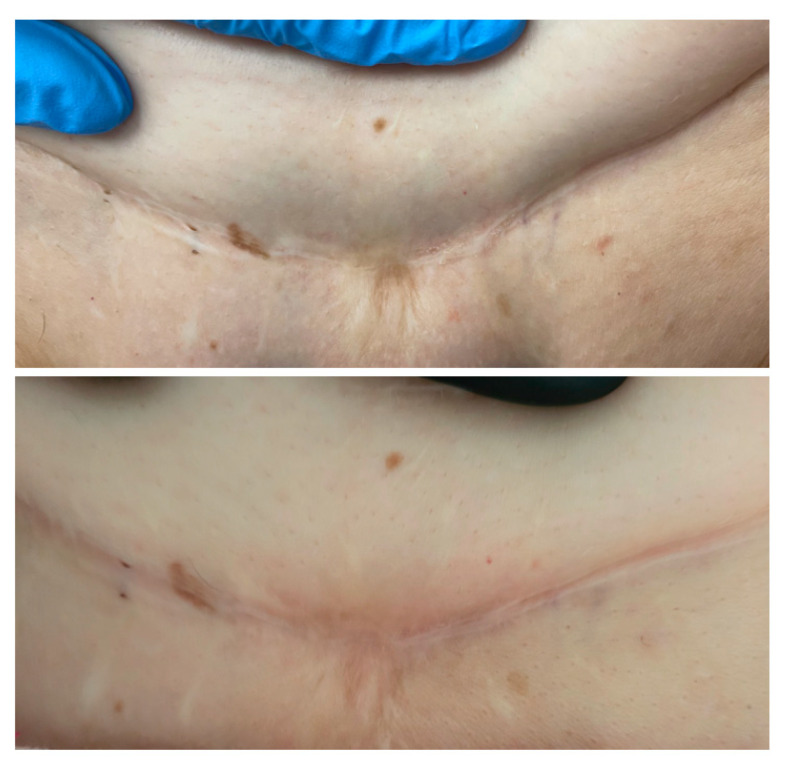

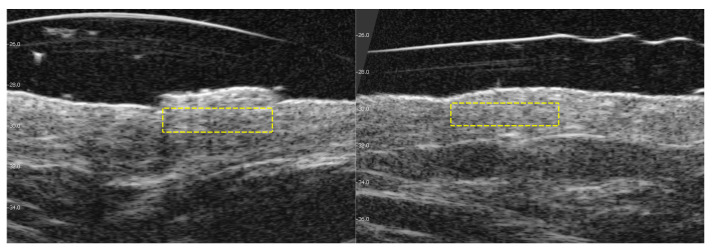
Visible ((**upper**) photo) and ultrasonographic ((**lower**) photo) changes before (**left**) and after (**right**) scar therapy of patient P5.

**Figure 11:**
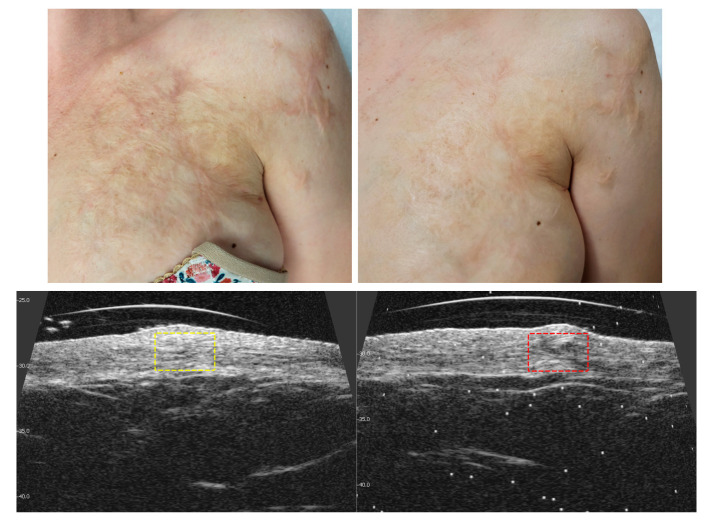
Visible ((**upper**) photo) and ultrasonographic ((**lower**) photo) changes before (**left**) and after (**right**) scar therapy of patient P6.

**Figure 12:**
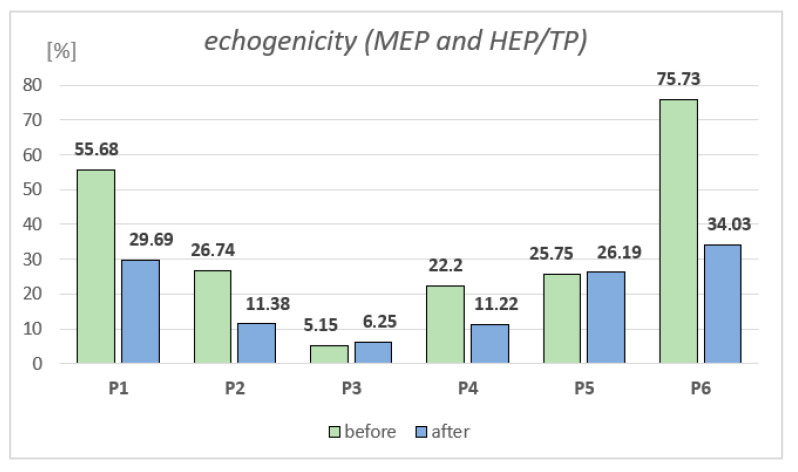
Differences in echogenicity of scars of studied patients.

Figure 6, Figure 7, Figure 8, Figure 9, Figure 10 and Figure 11 present the visible effects and ultrasound images showing changes in echogenicity before and after treatment among patients. Following the measurements of skin echogenicity (Figure 12) in the scar areas of the patients, it was observed that in four cases (P1, P2, P4, and P6), there was a reduction in the ratio of MEP and HEP (bright pixels) to the total number of pixels (TP). For patient P1 (scar on the hand), the pre-treatment ratio of bright pixels to the total number of pixels was 55.68%, which decreased to 29.69% after therapy. In the case of the scar after oncological port placement in patient P2, echogenicity decreased from 26.74% to 11.39% following the treatment. The echogenicity of scar in P4 decreased from 22.20% to 11.22%. 

## 4. Discussion

The presence of scars can significantly affect a person’s quality of life. Scars are often perceived by society as unattractive and unesthetic. They can have a considerable impact on both the physical and mental health of individuals. These imperfections are associated with sensations such as pain, itching, or tightness. Scar treatment is a time-consuming and prolonged process that does not always lead to success. There are tools available to assess the severity of scars; however, clinical management is mainly based on subjective scar assessment scales, which often demonstrate poor reliability. Currently, the clinical evaluation of scars still depends on subjective and error-prone scar rating scales, which hinder confident clinical decision-making. Although these scales, including the POSAS, are generally considered cost-effective and user-friendly, studies have shown that their accuracy is highly dependent on the examiner’s level of experience. Consequently, inter-rater reliability among clinicians remains limited [21,27]. Technology-based scar assessment tools, including the Cutometer^®^ and ultrasonography, which are being increasingly utilized in scar research, offer a potential solution by enabling the objective evaluation of scar tissue characteristics. Such tools may facilitate the early identification of scars at risk of hypertrophic development, thereby allowing for the timely and targeted implementation of therapeutic and rehabilitative interventions. It is clear that comprehensive scar assessment is essential for achieving the best possible outcomes in scar reduction.

One of the tools used to determine the type of scar is the POSAS questionnaire. This scar assessment scale is divided into two parts: the objective section completed by a clinician, and the subjective part assessed by the patient. In this study, based on the results from the POSAS questionnaire, high effectiveness of scar reduction treatments was demonstrated, which was reflected in changes in the scores provided by patients. The most significant improvements were observed in parameters such as color, stiffness, thickness, and irregularity. The POSAS questionnaire proved useful in a study conducted by Kurup et al. [28] during the treatment of post-burn hypertrophic scars using ablative fractional lasers combined with medication. The authors demonstrated that pigmentation and color, as measured by the POSAS, improved after just two treatment sessions in the responsive group and after two or three sessions in the non-responsive group.

The usefulness of the POSAS questionnaire was also evaluated in a study comparing scar tissue resulting from incisions made using monopolar electrosurgery and a scalpel. Scars were assessed 3 months post-surgery. Based on the POSAS questionnaire, the authors found no significant differences between scars resulting from the two methods. Moreover, the evaluations from both patients and researchers were similar, confirming POSAS as a reliable tool for scar assessment [29]. Other studies have also confirmed the high usefulness and reliability of the POSAS results, making it a widely used tool in scar assessment research [30,31].

The Cutometer is a commonly used device in scientific publications for scar assessment. It allows for the measurement of skin elasticity before and after treatment. In the present study, improved scar elasticity was observed in all patients. As is consistent with the previous literature, our results support the use of the output parameter R0 as a reliable and clinically relevant measure of scar firmness and pliability in the functional assessment of scars [13,22]. The best results were seen in patients P1 and P3, with increases of 378% and 176%, respectively. Patient P2 showed the least improvement, with a 32% increase, most likely because their scar initially exhibited the highest baseline elasticity among all participants. Furthermore, according to the POSAS, the patient self-assessed the scar thickness at a level of 4.

Nedelec et al. [32] studied the effects of massage on post-burn scars in a group of 60 individuals who received massages over their scars for 12 weeks. For each patient, two scars were selected: one was massaged and treated daily, while the control scar received only daily care. Measurements were taken before and after the massage sessions using the Cutometer. The results showed that elasticity improved by week 8 in the massaged scars and by week 10 in the control scars. Scar thickness also decreased—by week 5 in the control scars and by week 7 in the massaged ones. Although the authors did not find significant differences between the two types of scars, the Cutometer proved to be an effective and objective tool for measuring elasticity and comparing outcomes. Lubczyńska et al. [33] showed a significant increase in skin elasticity in scarred areas in 11 individuals undergoing combined therapy techniques such as massage, dry needling, and taping. Sano et al. [34] examined the extensibility and elasticity of human skin at different body locations and how these properties influence scarring. Measurements were taken at 16 body sites in five participants using the Cutometer MPA 580 (Courage + Khazaka). The study showed that the least elastic skin is located on the earlobes, upper eyelids, palms, and soles, while the most stretchable areas are the abdomen, scapular region, and deltoid muscle.

Ultrasound imaging enables the measurement and visualization of both healthy and affected skin structures. In the present study, improvement was observed in four out of six patients. When assessing scars, echogenicity of the skin is analyzed. Hyperechogenic areas (bright pixels) represent collagen fibers, which are often abnormally structured in scars and appear as bright zones. Patients P1, P2, P4, and P6 showed a reduction in bright pixels relative to the total pixels (TP). In our study, no substantial change in echogenicity was observed in patient P3, despite a noticeable improvement in elasticity (Cutometer^®^ measurement) as well as visible scar reduction. This finding may be explained by the fact that patient P3 presented with the most hypertrophic scar among all study participants. It is important to note that the 48 MHz ultrasound probe used in this study has an optimal focal depth of approximately 3–4 mm. As a result, in the ultrasound image (Figure 9), a lower echogenicity band is visible beneath the elevated area of the scar, likely reflecting the structural characteristics of deeper scar tissue beyond the primary focus range of the transducer. Satpathy et al. [35] compared postoperative scar imaging using ultrasound and magnetic resonance imaging (MRI). The study involved 30 women with cesarean scars. Both imaging methods measured scar thickness before and after the incision. Bland–Altman analysis revealed minor errors in both methods, but ultrasound proved to be more accurate, with a precision of 96.7%, compared to approximately 90% for MRI. The results from both devices were comparable. Malinowska et al. [19] conducted a study on the reduction in acne scars using laser therapy. The DermaMed ultrasound device was used to measure scar parameters before and after treatment. The study involved seven patients aged 29 to 43 with facial acne scars. Significant improvements were noted in epidermal thickness (0.13 mm before and 0.11 mm after), depth (0.36 mm before and 0.29 mm after), and width (2.43 mm before and 2.35 mm after). No significant changes were observed in dermis thickness (1.22 mm before and 1.23 mm after).

Various methods are currently used to reduce and improve the appearance of scars. These include laser therapy, chemical peels, scar mobilization techniques, skin care routines, microneedling, and skin needling. Depending on the type and severity of the scar, different reduction methods are applied, but the best results are often achieved by combining several approaches.

Chemical peels, such as trichloroacetic acid (TCA), are widely used in scar treatment. The concentration of TCA varies depending on the scar’s size and depth. Sun and Lim [36] published studies on the use of TCA for atrophic and polymorphic acne scars. In their study of 41 participants, each underwent three treatment sessions with 90% TCA applied to the scar area. The results were highly satisfactory: 41% improvement in 9 patients, and 59% in 13 individuals. Two patients experienced side effects, and five were taking isotretinoin concurrently, though this did not affect reepithelialization time or cause adverse effects.

Identifying the scar type and characteristics enables faster and more effective therapy selection. Understanding the anatomy of the defect allows for optimal laser choice and creation of an individualized treatment plan for each patient, which may involve both surgical and non-surgical interventions [37]. Nobari et al. [38] compared the effectiveness of fractional needle lasers (radiofrequency, mesoneedling, microneedling) with ablative lasers (CO_2_, YAG) in treating atrophic and hypertrophic scars. Both methods showed excellent results, with approximately 70% of patients reporting improvement. The difference in effectiveness between the two approaches was negligible. Recently it has been shown that the combination of a CO_2_ laser with 1540/1570 nm wavelengths constitutes a promising approach for scar treatment, targeting scar tissue remodeling while minimizing patient downtime [39].

One of the least invasive methods is scar mobilization therapy. Manual therapy can reduce pain, itching, stiffness, size, and color and improve scar flexibility. It also has a positive effect on skin parameters such as elasticity (measured with the Cutometer) and thickness. The most common type of post-burn scar is a hypertrophic scar. Lin et al. [40] conducted a study involving 420 individuals with this scar type. Each received scar mobilization techniques, including massage, for 12 weeks. Participants attended sessions one to three times per week, lasting 5 to 30 min. The results showed improvements in scar thickness and reductions in pain, itching, and social anxiety. Pain, itchiness, and anxiety symptoms significantly decreased.

Microneedling techniques are increasingly used for scar treatment. Skin needling is a modern, effective method with minimal side effects. In this study, all six participants who underwent three sessions at intervals of 4 to 6 weeks showed significant improvement—reduced prominence, surface area, and discoloration and lightening of scars. Some (P1, P2, P4, and P5) had adhesions that were resolved. Villani et al. [41] conducted a literature review on microneedling combined with skin needling. Articles were retrieved from databases such as PubMed, Medline, and Embase. Almost all reviewed studies confirmed the method’s positive effects. Even better results were achieved when combining microneedling with lasers, chemical peels, platelet-rich plasma (PRP), or fillers. Lew et al. [42] compared manual therapy and dry needling for neck and upper back pain. Six studies involving 240 participants were analyzed using articles from PubMed, PEDro, and CINAHL from the last 10 years. The results showed that both techniques reduced pain and improved function, with only minimal differences between them. Alam et al. [43] studied the effectiveness of microneedling in acne scar therapy. Fifteen adult patients underwent three microneedling treatments on one side of the face, spaced two weeks apart. Photographs were taken before and three and six months after treatment. Two independent dermatologists positively evaluated the results. The greatest improvement was seen between baseline and month six. Minimal changes were observed between months three and six. The control group showed no significant changes. On average, patients reported a 41% improvement in scar appearance. No side effects were reported, and pain levels averaged 1.08 out of 10.

It should be taken into consideration that differences in the obtained measurements and the effects observed in ultrasound images as well as photographs depend on the location of the scars and the age of each scar. Limitations of this study include the small study group, the absence of a control group, and the various types of scars.

## 5. Conclusions

Based on the results of this study and a review of the literature, it can be concluded that many effective methods for scar therapy are currently available. The skin needling technique is an effective method for scar treatment, and the risk of adverse effects or complications after a series of procedures is minimal. Skin needling supported by adequate scar care with a topical product throughout the treatment period is an effective method for reducing various types of scars, particularly in terms of surface leveling decreasing protrusion and thickness and increasing elasticity. The POSAS questionnaire is a useful tool for assessing the patient’s subjective perception of their scar and for comparing the effects of the skin needling therapy. High-frequency ultrasonography and Cutometer examination enable an objective assessment of the scar area and allow for a comparison of treatment effectiveness. Further research is needed to determine whether our findings are applicable to the longitudinal assessment of scars.

## Figures and Tables

**Table 1:** Definition of Cutometer output parameters.

R-Parameter	Absolute Parameter	Definition
R0	=Uf	Maximum skin deformation [mm]
R1	=Uf Ua	Difference max. deformation/final retraction
R2	=Ua/Uf	Rational retraction/max. deformation
R3	=last max. amplitude	If repeated circles of negative pressure/release are applied
R4	=last min. amplitude	If repeated circles of negative pressure/release are applied
R5	=Ur/Ue	Ration immediate retraction/immediate deformation
R6	=Uv/Ue	Rationale deformation/immediate deformation
R7	=Ur/Uf	Ratio immediate retraction/maximum deformation
R8	=Ua	Final retraction [mm]

**Table 2:** The characteristics of the participants and their scars.

Patient Number	Age	Scar Type	Location	Time Since Injury	Previous Treatment
P1	30	Post-traumatic	Dorsal side of hand	1.5 years	None
P2	56	Post-surgical	Chest	5 years	None
P3	37	Post-surgical	Lower abdomen	1.5 years	None
P4	34	Post-surgical	Neck	2.5 years	None
P5	49	Post-surgical	Lower abdomen	42 years	Physiotherapeutic mobilization
P6	35	Post-burn	Chest	32 years	None

**Table 3:** POSAS patient and observer scale results.

POSAS Patient Scale Results														
Category	P1		P2		P3		P4		P5		P6		Mean Score	
	Before	After	Before	After	Before	After	Before	After	Before	After	Before	After	Before	After
Pain	1	1	3	1	3	1	1	1	1	1	4	1	2.2	1.0
Itchiness	3	3	2	1	8	2	1	1	1	1	2	2	2.8	1.7
Color	10	5	7	3	9	3	10	7	3	1	5	4	7.3	3.8
Stiffness	8	2	4	2	9	3	10	5	10	8	8	4	8.2	4.0
Thickness	8	2	4	2	8	3	10	7	10	10	10	3	8.3	4.5
Irregularity	7	3	8	4	8	3	10	7	10	9	10	4	8.8	5.0
Overall	10	3	8	4	8	4	10	7	10	9	9	5	9.2	5.3
TOTAL	47	19	36	17	53	19	52	35	45	39	48	23	46.8	25.3
**POSAS Observer Scale Results**														
**Category**	**P1**		**P2**		**P3**		**P4**		**P5**		**P6**		**Mean Score**	
	**Before**	**After**	**Before**	**After**	**Before**	**After**	**Before**	**After**	**Before**	**After**	**Before**	**After**	**Before**	**After**
Vascularity	8	6	5	4	8	5	10	8	1	1	4	3	6.0	4.5
Pigmentation	7	5	4	4	6	4	7	4	3	2	7	4	5.7	3.8
Thickness	5	3	5	2	5	4	7	5	5	4	4	2	5.2	3.3
Relief	7	3	4	2	7	5	6	5	5	4	6	3	5.8	3.7
Pliability	5	3	5	3	8	6	5	4	8	7	8	3	6.5	4.3
Surface area	3	2	6	3	8	5	9	6	3	3	6	3	5.8	3.7
Overall	6	4	6	3	7	5	7	5	7	6	8	4	6.8	4.5
TOTAL	41	26	35	21	49	34	51	37	32	27	43	22	41.8	27.8

## Data Availability

The original contributions presented in the study are included in the article; further inquiries can be directed to the corresponding author.
